# Corps étranger métallique inhalé: 36 mois d’évolution intrabronchique chez un enfant de 8 ans

**DOI:** 10.11604/pamj.2014.18.225.4823

**Published:** 2014-07-17

**Authors:** Alexis Kumba Mupepe, Olivier Mukuku, Yves Bagale, Bienvenu Mukuku Ruhindiza

**Affiliations:** 1 Institut Supérieur des Techniques Médicales d'Uvira, République Démocratique du Congo; 2 Hôpital Général de Référence d'Uvira, République Démocratique du Congo; 3 Faculté de Médecine, Université de Lubumbashi, République Démocratique du Congo; 4 Faculté de Médecine, Université de Ngozi, Burundi

**Keywords:** Inhalation, corps étranger, intrabronchique, evolution, Inhalation, foreign body, intrabronchial, evolution

## Abstract

Les corps étrangers dans les voies respiratoires constituent une urgence relativement fréquente chez les enfants. Nous présentons ici le cas d'une fillette de 8 ans qui avait inhalé un clou que la radiographie avait localisé dans la bronche droite. L’évolution est marquée par une symptomatologie muette et une migration vers le bas de ce clou. Ne disposant pas d’équipement pour une chirurgie adaptée, le malade vit encore avec ce corps étranger en lui. La nature et l’évolution clinique et paraclinique rapportées ici en constituent l'intérêt.

## Introduction

Les corps étrangers (CE) dans les voies respiratoires constituent une urgence relativement fréquente chez les enfants. Ils sont l′apanage des enfants délaissés, ne faisant l′objet d′aucune surveillance parentale. Selon les données de la littérature, on note une nette prédominance masculine et l′âge de prédilection se situe entre 1 et 3 ans [1–3]. Cette pathologie est fréquente et sa gravité revêt des degrés très variables. En fait, le CE peut être un incident totalement anodin ou être contraire responsable de complications graves, parfois vitales. Leur diagnostic est le plus souvent facile et la prise en charge obéit à une règle générale selon laquelle tout CE ayant pénétré par les voies naturelles peut être extrait par les mêmes voies [4]. Nous présentons ici le cas d′une fillette de 8 ans qui avait inhalé un clou que la radiographie avait localisé dans la bronche droite. L′intérêt de cette observation réside dans la particularité du type de CE, de la difficulté de prise en charge dans un milieu sous-équipé comme le nôtre ainsi que de son évolution.

## Patient et observation

**Figure 1:**
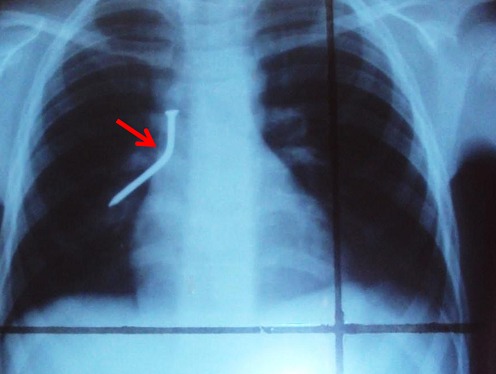
Radiographie du thorax vue de face montrant le clou dans la bronche droite quelques heures après inhalation (à l'admission)

**Figure 2:**
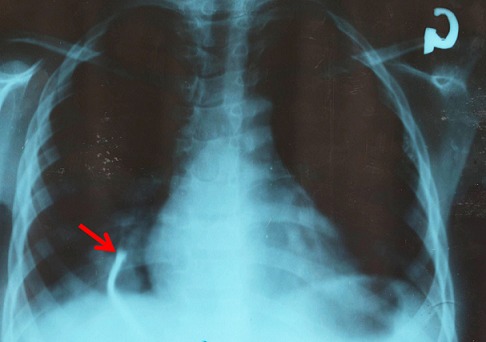
Radiographie du thorax vue de face montrant la progression du clou dans la bronche droite vers le bas 36 mois après son inhalation

Une fille de 8 ans, est admise aux urgences de l′hôpital général de référence d′Uvira (à l′Est de la République démocratique du Congo) pour avoir avaler un objet métallique (clou). Pendant que tout le monde était absent de la maison, l′enfant jouait avec un clou dans sa bouche. Par mégarde, le clou traverse le pharynx, elle se met à crier et l′entourage l′emmènera directement à l′hôpital. L′examen clinique montre un bon état général, un bon état hémodynamique mais une importante agitation. Pas de détresse respiratoire, pas de dysphagie, pas de douleur, pas de fièvre mise en évidence. L′auscultation relève des râles sibilants du côté droit. La radiographie thoracique fait état d′un CE à type d′opacité longitudinale à l′hémi-thorax droit (Figure 1). Plusieurs clichés radiographiques avaient été réalisés à 2, 6, 12 et 24 heures après le premier cliché et ils avaient été identiques au premier. L′hôpital ne disposant pas de matériel de bronchoscopie, nous avons juste fait une endoscopie haute pour éliminer toute effraction du clou à travers l′oesophage. Douze mois après, l′image radiologique ne montrait aucune différence avec celles prises à son admission. Trente-six mois plus tard après l′inhalation du clou, l′image radiographique prise montre que le clou avait migré vers le bas (Figure 2) et l′examen clinique ne rapporte aucune particularité. Ne disposant pas d′équipement pour une chirurgie adaptée, le malade vit encore avec ce corps étranger en lui.

## Discussion

Les CE retrouvés en ORL sont de nature diverse (cacahuète, hameçon, morceau de bois, bouchon de stylo, dent, etc.) et de tous les CE extraits dans les voies aéro-digestives, le clou est rarement retrouvé [3–6]. Les circonstances de survenue d′inhalation d′un corps étranger chez les enfants sont souvent accidentelles chez les enfants; l′objet introduit dans la bouche est inhalé fortuitement à l′occasion d′un choc ou d′un effet de surprise comme s′était le cas dans notre observation, l′enfant avait été surprise en train de jouer avec les clous par ses parents. Comparée la bronche souche gauche, la bronche souche droite, en raison de son obliquité (les angles bronchiques avec l′axe trachéal sont de 30° à droite et de 45° à gauche) et de son calibre légèrement supérieur, est le siège préférentiel du CE [4, 7]. Par contre, dans certaines séries, les auteurs rapportent des fréquences élevées de CE dans la bronche souche gauche que celle de droite [3, 8].

Sur le plan clinique, l′irruption dans les voies aériennes d′un CE est toujours marquée par un syndrome clinique de grande valeur diagnostique: le syndrome de pénétration qui est marqué par la survenue brutale d′un accès de suffocation suivi immédiatement de quintes de toux expulsives puis d′un tirage inspiratoire entre les quintes. L′auscultation met en évidence un wheezing, des râles sibilants ou des sous-crépitants unilatéraux [2, 6, 9]. Dans notre observation, la symptomatologie était presque muette, seuls des râles sibilants étaient notés lors de notre examen clinique. Ceci serait tout simplement expliqué par l′écart de temps entre l′incident et l′admission à l′hôpital car dans la plupart des cas, tout rentre dans l′ordre en quelques minutes [2]. La bronchoscopie reste l′examen de choix mais notre plateau technique souffre du sous-équipement et de la sous-médicalisation. Celle-ci permet non seulement de visualiser le CE, mais aussi de l′extractaire sous contrôle de la vue à l′aide de la pince de Magill en obéissant à l′apophtegme de Chevalier Jackson qui dit que « tout CE des voies aéro-digestives qui a pénétré par les voies naturelles peut être extrait par les mêmes voies à condition qu′il n′ait pas migré à travers la paroi perforée de ces voies » [3, 4]. L′évolution d′un CE intrabronchique passe par trois stades évolutifs décrits à l′endoscopie: le premier stade correspondant à la réaction bronchique locale de type inflammatoire; le deuxième étant celui de la réaction granulomateuse créant un granulome végétant endoluminal, responsable de l′enclavement du CE dans la lumière bronchique; et le dernier est un stade tardif, où les lésions bronchiques sont intenses; l′inflammation gagne les tissus péribronchiques et les ganglions de voisinage et les CE sont invisibles à la fibroscopie [5]. Le séjour prolongé du CE dans la bronche expose à un risque infectieux secondaire à l′oedème, l′hypersécrétion réactionnelle et la stase vasculaire qui se créent et au fil du temps une suppuration peut s′installer. D′où sa négligence ou sa méconnaissance peut être à l′origine de dégâts parenchymateux parfois irréversibles, imposant des exérèses pulmonaires étendues [5]. Dans notre cas, nous n′avons observé aucune complication malgré trente-six mois d′évolution. Notre cas semble être une particularité, ainsi des auteurs ont rapporté d′infections bronchopulmonaires trainantes ou récidivantes, des hémoptysies, un pneumothorax, un emphysème [5, 10]. Dans sa série de 47 cas de CE trachéobronchiques répertoriés, Adebayo décrit celui d′une fillette de 4 ans présentant une récurrente toux productive purulente et une fièvre chronique suite à une rétention du clou dans la bronche droite pendant 14 semaines. Après échec d′extraction bronchoscopique, une bronchotomie avait permis d′extraire le clou [10].

## Conclusion

L'inhalation accidentelle de CE constitue une cause de morbidité et de mortalité chez les enfants. Les patients doivent bénéficier d'une prise en charge immédiate et adéquate. D'où la nécessité de prévenir ces accidents chez les enfants par une éducation du milieu familial. Le présent cas que nous avons rapporté est particulier par sa longue durée asymptomatique au niveau de la bronche droite.
